# Molecular and Pharmacological Characterization of β-Adrenergic-like Octopamine Receptors in the Endoparasitoid *Cotesia chilonis* (Hymenoptera: Braconidae)

**DOI:** 10.3390/ijms232314513

**Published:** 2022-11-22

**Authors:** Gang Xu, Yuan-Yuan Zhang, Gui-Xiang Gu, Guo-Qing Yang, Gong-Yin Ye

**Affiliations:** 1 College of Plant Protection, Yangzhou University, Yangzhou 225009, China; 2 State Key Laboratory of Rice Biology & Ministry of Agricultural and Rural Affairs Key Laboratory of Molecular Biology of Crop Pathogens and Insects, Institute of Insect Sciences, Zhejiang University, Hangzhou 310058, China

**Keywords:** octopamine receptor, parasitoid, expression profiles, cAMP, pharmacology

## Abstract

Octopamine (OA) is structurally and functionally similar to adrenaline/noradrenaline in vertebrates, and OA modulates diverse physiological and behavioral processes in invertebrates. OA exerts its actions by binding to specific octopamine receptors (OARs). Functional and pharmacological characterization of OARs have been investigated in several insects. However, the literature on OARs is scarce for parasitoids. Here we cloned three β-adrenergic-like OARs (*CcOctβRs*) from *Cotesia chilonis*. CcOctβRs share high similarity with their own orthologous receptors. The transcript levels of *CcOctβRs* were varied in different tissues. When heterologously expressed in CHO-K1 cells, CcOctβRs induced cAMP production, and were dose-dependently activated by OA, TA and putative octopaminergic agonists. Their activities were inhibited by potential antagonists and were most efficiently blocked by epinastine. Our study offers important information about the molecular and pharmacological properties of β-adrenergic-like OARs from *C. chilonis* that will provide the basis to reveal the contribution of individual receptors to the physiological processes and behaviors in parasitoids.

## 1. Introduction

Octopamine (OA) is a biogenic monoamine acting as a neurohormone, neuromodulator, and neurotransmitter in invertebrate physiology and behaviors [1]. Norepinephrine and OA are similar in structure and function, and they are generally considered counterparts in vertebrates and invertebrates, respectively [2,3]. OA is abundant in the central nervous system of insects [4,5], and regulates diverse processes of physiology and behaviors, such as feeding [6,7,8], aggression [9,10,11,12], sleep [13,14], courtship [15,16], post-mating [17,18], oviposition [19,20,21], learning [22,23], reward [24], immunity [25,26], exercise adaption [27,28], thermogenesis [29], and muscle contractility [30].

OA exerts its activities via binding to membrane proteins OA receptors (OARs) that belong to the class A (rhodopsin-like) G protein-coupled receptor (GPCR) family [31]. Insect OARs can be classified into three major groups based on their structural, pharmacological and biochemical properties, similar to vertebrate α-adrenergic or β-adrenergic receptors: α_1_-adrenergic-like OARs (Octα1R, also referred to as OAMB), β-adrenergic-like OARs (OctβR), and α_2_-adrenergic-like OARs (Octα2R) [32,33,34]. Activation of Octα1R can elevate intracellular Ca^2+^ levels at lower concentrations of OA as compared to increasing cyclic adenosine monophosphate (cAMP) levels, whereas activation of OctβRs selectively stimulate intracellular cAMP production [35,36]. Octα2R has two alternative splicing forms, and they are both activated by OA, resulting in the inhibition of forskolin-simulated intracellular cAMP levels [33].

*Cotesia chilonis* (Hymenoptera: Braconidae) is an obligate larval endoparasitoid that plays a key role in regulating the population density of *Chilo suppressalis* (Lepidoptera: Crambidae), which is one of the most economically important rice pests in China and other Asian countries [37,38,39]. Investigating the properties of OARs can contribute to understand the potential functions of octopaminergic systems in insects. Although the β-adrenergic-like OAR family has been characterized in *Drosophila melanogaster* [40], *Apis mellifera* [41], *Nilaparvata lugens* [42], and *Plutella xylostella* [43], the molecular information, expression patterns and pharmacological properties of the β-adrenergic-like OAR family from *C. chilonis* remain poorly known. The β-adrenergic-like OAR family generally has three subtypes, including Octβ1R, Octβ2R, and Octβ3R. Octβ1R regulates feeding and learning behavior [23,44], and Octβ3R plays an essential role in metamorphosis regulation of ecdysone synthesis and pupation [45,46], whereas Octβ2R has multiple functions, including ovulation [47,48,49], locomotion [50,51,52], and stress resistance [53].

In this study, we cloned three β-adrenergic-like OARs from *C. chilonis*, named *CcOctβ1R*, *CcOctβ2R*, and *CcOctβ3R*, respectively. Their expression profiles were analyzed, and further, their pharmacological properties were comprehensively investigated. This study will be an important step toward understanding the actions of OA in the behavior and physiology of parasitoids and provide a basis for enhancing the role of natural enemies in the manipulation of insect pests.

## 2. Results

### 2.1. Cloning and Sequence Analysis of CcOctβ1R, CcOctβ2R, and CcOctβ3R

The fragments of three putative β-adrenergic-like OARs were identified from the transcriptome data of *C. chilonis*. The full lengths of cDNA of *CcOctβ1R*, *CcOctβ2R*, and *CcOctβ3R* were amplified by RT-PCR and further verified via DNA sequencing. The sequences of *CcOctβRs* were submitted to the GenBank with the following accession numbers: *CcOctβ1R* (OP422531), *CcOctβ2R* (OP422532), *CcOctβ3R* (OP422533). The deduced amino-acid sequences contain 417, 435, and 358 residues for CcOctβ1R, CcOctβ2R, and CcOctβ3R, respectively.

Amino acid sequence comparisons showed the sequence identities and similarities of Octβ1R, Octβ2R, Octβ3R between *C. chilonis* and other insects. CcOctβ1R shares high identities and similarities with AmOctβ1R (75%, 84%), NlOctβ1R (57%, 64%), CsOctβ1R (55%, 68%), TcOctβ1R (48%, 58%), and DmOctβ1R (42%, 56%) (Supplementary Figure S1). The closest relationship occurs between CcOctβ2R and AmOctβ2R with 67% identity and 77% similarity, followed by TcOctβ2R (63%, 76%), NlOctβ2R (63%, 75%), CsOctβ2R (53%, 65%), and DmOctβ2R (46%, 58%) (Supplementary Figure S2). The identities and similarities of CcOctβ3R compared to other Octβ3R orthologues are 69% and 76% for AmOctβ3R, 55% and 66% for NlOctβ3R, 55% and 66% for TcOctβ3R, 40% and 49% for DmOctβ3R (Supplementary Figure S3).

Sequence alignments of three types of β-adrenergic-like OARs showed the typical characteristic GPCR features, including seven transmembrane (TM1-TM7) regions, an extracellular N-terminus, an intracellular C-terminus, and potential phosphorylation sites by protein kinase C (PKC) ([S/T]-x-[R/K]), as well as potential N-glycosylation sites (N-x-[S/T]) (Supplementary Figures S1–S3). The motifs (Asp residue in TM3, Ser residue in TM5 and Tyr residue in TM6) essential for receptor activation, ligand binding, and G-protein coupling typical for OARs are well conserved in CcOctβ1R, CcOctβ2R, and CcOctβ3R (Supplementary Figures S1–S3). The consensus sequence F^6.44^-X-X-C^6.47^-W^6.48^-X-P^6.50^-F^6.51^-F^6.52^ (F_306_LACWLPFF in CcOctβ1R, F_319_ILCWLPFF in CcOctβ2R, and F_279_LLCWLPFF in CcOctβ3R) is conserved in TM6 (Supplementary Figures S1–S3), and two Phe residues (F^6.51^-F^6.52^) are unique to aminergic receptors.

### 2.2. Phylogenetic Analysis of CcOctβRs

**Figure 1:**
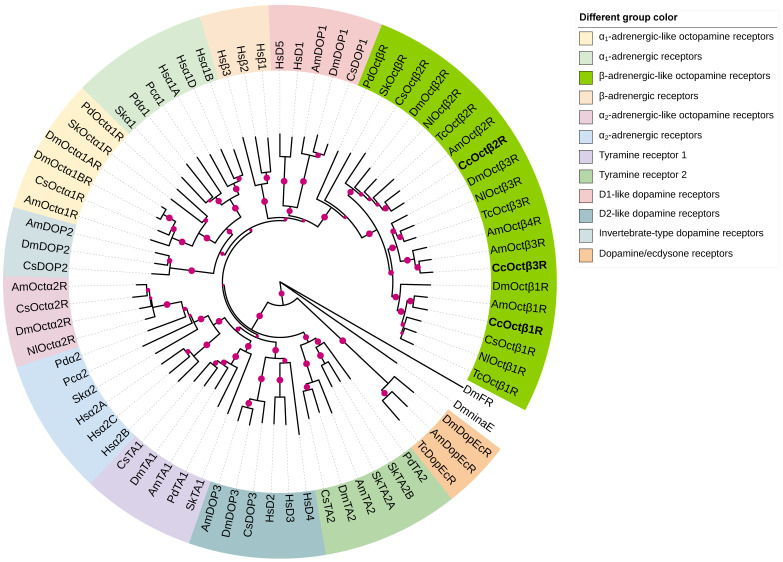
Phylogenetic relationship of CcOctβRs and various biogenic amine receptors. MEGA 7.0 software was used to construct the neighbor-joining tree. *Drosophila* ninaE rhodopsin 1 (CG4550) and FMRFamide receptor (CG2114) were used as outgroups. CcOctβRs are in bold. The accession numbers of amino acid sequences used in the phylogenetic analysis were indicated in Supplementary Table S1. Abbreviations: Am, *Apis mellifera*; Dm, *Drosophila melanogaster*; Tc, *Tribolium castaneum*; Cs, *Chilo suppressalis*; Nl, *Nilaparvata lugens*; Hs, *Homo sapiens*; Pc, *Priapulus caudatus*; Pd, *Priapulus caudatus*; Sk, *Saccoglossus kowalevskii*. Hs and Sk are deuterostomian species, and the others are protostomian species.

In order to characterize the evolutionary relationships between β-adrenergic-like OARs of *C. chilonis* and other biogenic amine receptors, as well as provide more information about their potential functional roles, the phylogenetic analysis was performed with CcOctβRs and biogenic amine receptors from protostomian and deuterostomian species (Figure 1 and Supplementary Table S1). The phylogenetic tree indicated that CcOctβRs assembled in a clade that contains β-adrenergic-like OARs from *A. mellifera* (AmOctβ1-4R), *Tribolium castaneum* (TcOctβ1-3R), *N. lugens* (NlOctβ1-3R), *D. melanogaster* (DmOctβ1-3R), *C. suppressalis* (CsOctβ1-2R), *Priapulus caudatus* (PcOctβR), and *Saccoglossus kowalevskii* (SkOctβR). This clade clustered nicely with D1-like DARs and human β-adrenergic receptors (Figure 1). In contrast, Octα1Rs were closely related to α_1_-adrenergic receptors and invertebrate-type DARs (Figure 1). Octα2Rs formed a sister group with α_2_-adrenergic receptors and tyramine receptor 1 (Figure 1).

### 2.3. Expression Profiles of CcOctβRs

**Figure 2:**
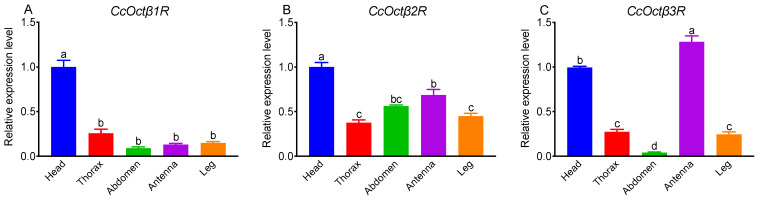
Relative transcript levels of *CcOctβ1R* (**A**), *CcOctβ2R* (**B**), and *CcOctβ3R* (**C**) in different tissues of *Cotesia chilonis* adults. Different lowercase letters on the bars represent statistical differences in the transcript levels (*p* < 0.05, Tukey’s HSD test). Data are expressed as the means ± SE of three biological replicates.

The expression profiles of *CcOctβ1R*, *CcOctβ2R*, and *CcOctβ3R* were investigated in different tissues of *C. chilonis*, including head, thorax, abdomen, antenna, and leg. qRT-PCR results showed that the relative expression levels of *CcOctβ1R*, *CcOctβ2R*, and *CcOctβ3R* were varied in various tissues. *CcOctβ1R* and *CcOctβ2R* were mostly expressed in the head, while *CcOctβ3R* was more highly expressed in the antenna than in the head (Figure 2). *CcOctβ2R* displayed a relatively higher expression in the antenna than abdomen, leg and thorax (Figure 2B), and the transcript level of *CcOctβ3R* in the thorax and leg was higher than in the abdomen (Figure 2C).

### 2.4. Ligand Specificity of CcOctβRs

**Figure 3:**
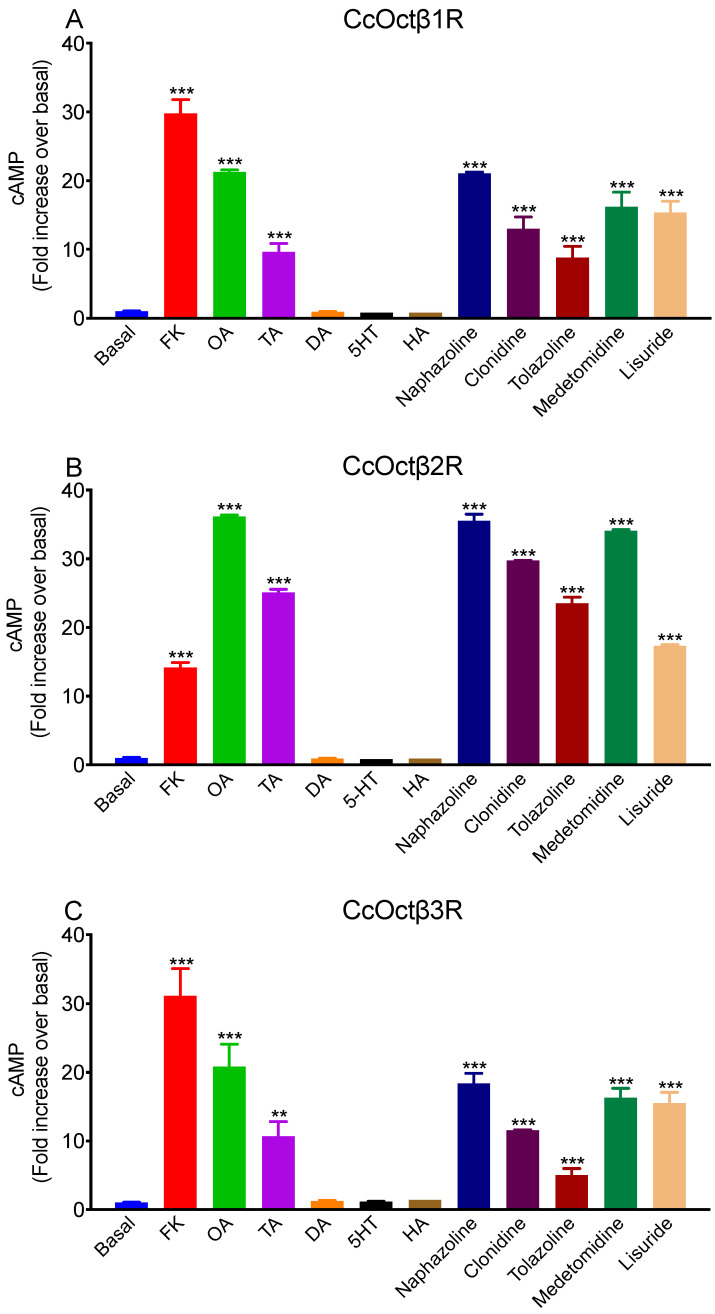
Effects of various biogenic amines and putative synthetic agonists on intracellular cAMP production in CcOctβ1R (**A**)-, CcOctβ2R (**B**)-, and CcOctβ3R (**C**)-expressing CHO-K1 cells. Biogenic amines and agonists were measured with a concentration of 1 μΜ, and forskolin (10 μM) served as a positive control. The statistical differences between the basal value and the treatments are indicated by the asterisks (** *p* < 0.01, *** *p* < 0.001, Dunnett’s multiple comparison test). Data are presented as means ± SE of four experiments.

To unravel second messenger coupling and pharmacological profiles of CcOctβ1R, CcOctβ2R, and CcOctβ3R, we obtained CHO-K1 cells stably expressing each receptor, respectively. The cAMP levels were determined in the cells when incubated in the presence of 1 μM concentrations of various biogenic amines and putative synthetic agonists including OA, TA, DA, 5-HT, HA, naphazoline, clonidine, tolazoline, medetomidine, and lisuride. Neither DA, 5-HT nor HA caused the elevation of cAMP production. In contrast, the cell lines of each receptor showed significantly increasing cAMP levels after application of OA, TA, naphazoline, clonidine, tolazoline, medetomidine and lisuride (Figure 3). Thus, we concluded that CcOctβ1R, CcOctβ2R, and CcOctβ3R couple to G_s_ protein.

**Figure 4:**
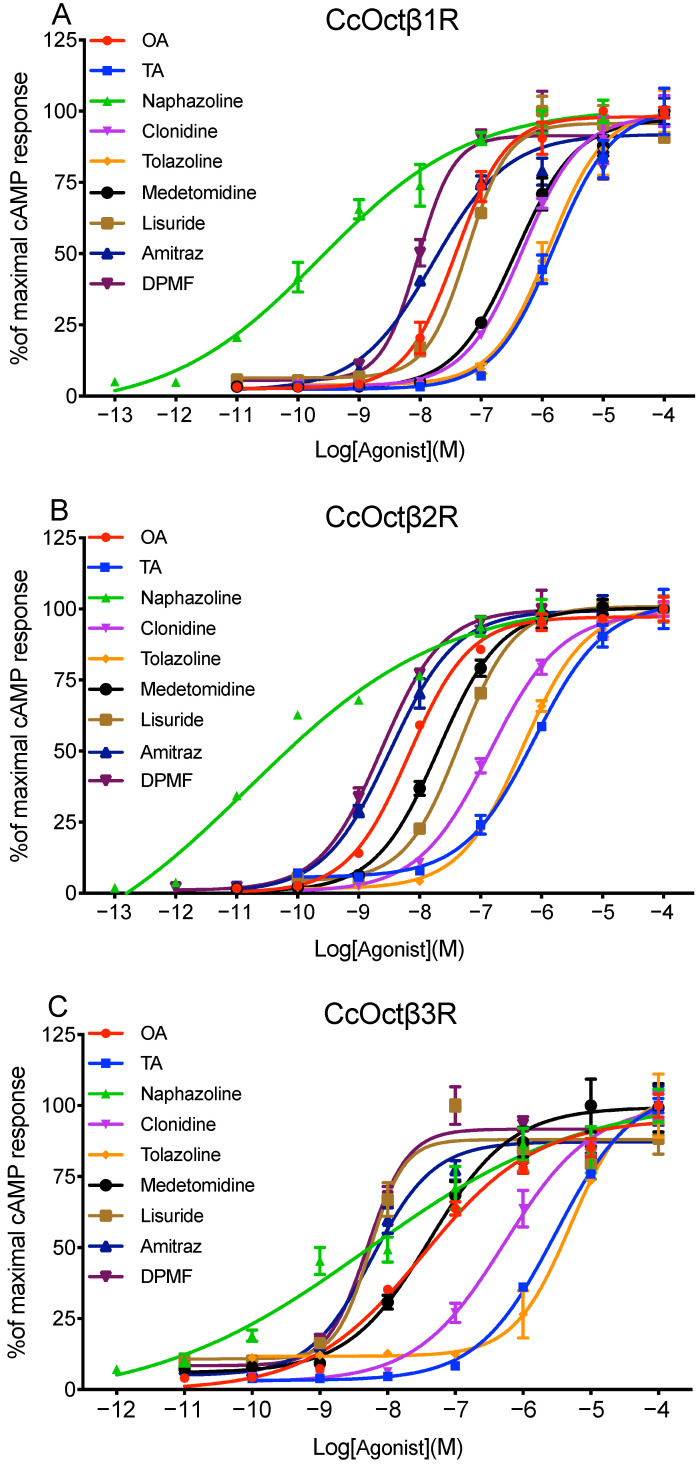
Concentration-response effects of OA, TA and various agonists on intracellular cAMP production in CcOctβ1R (**A**)-, CcOctβ2R (**B**)-, and CcOctβ3R (**C**)-expressing CHO-K1 cells. The values are calculated to the maximal cAMP response (=100%) for each agonist. Data are presented as means ± SE of four experiments.

Further, the increasing concentrations of OA, TA and seven potent synthetic agonists were applied to the cells, and dose-response curves for each receptor were displayed (Figure 4). The rank order of agonist potency for CcOctβ1R was naphazoline > DMPF > amitraz > OA > lisuride > medetomidine > clonidine > tolazoline > TA (Figure 4A, Table 1). Similarly, half-maximal stimulation (EC_50_) values for tested compounds were calculated from concentration-response curves for CcOctβ2R and CcOctβ3R (Figure 4B–C), and were summarized in Table 1. Half-maximal activations of CcOctβRs with OA, naphazoline, DMPF, amitraz and lisuride were in the low nanomolar range. OA was about two orders of magnitude more efficient than TA for CcOctβRs (Table 1).

### 2.5. Pharmacological Properties of CcOctβRs

**Figure 5:**
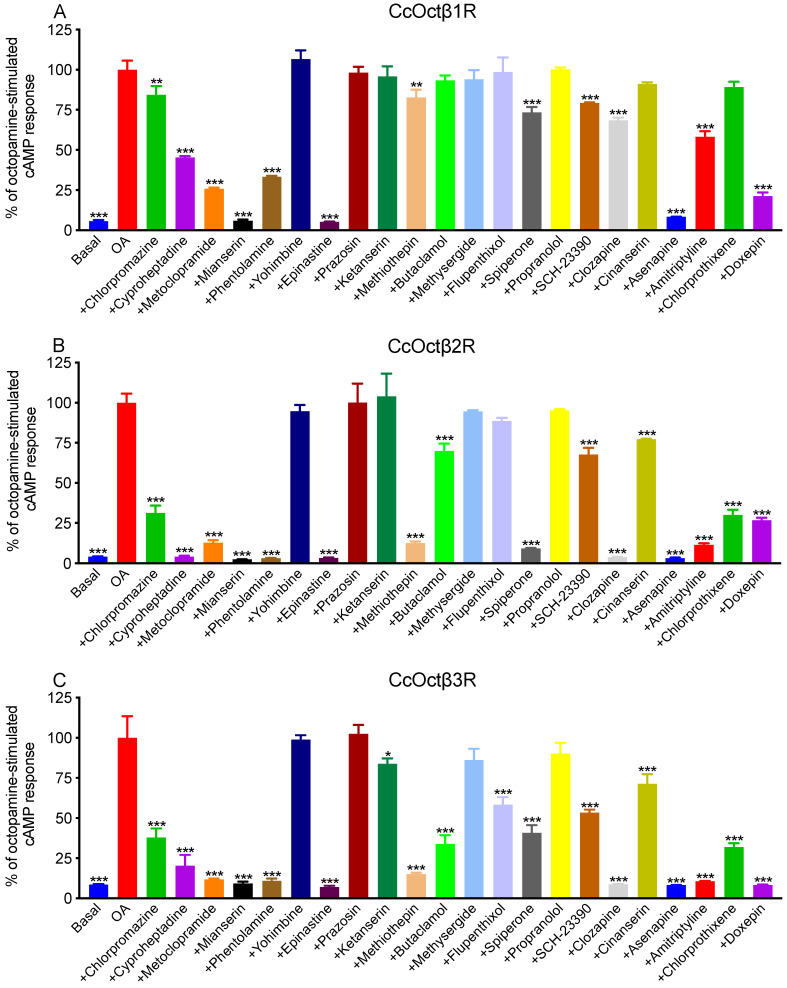
Effects of putative synthetic antagonists (10 μM) on OA-stimulated intracellular cAMP production in CcOctβ1R (**A**)-, CcOctβ2R (**B**)-, and CcOctβ3R (**C**)-expressing CHO-K1 cells. Dunnett’s multiple comparison test was performed for the statistical analysis (* *p* < 0.05, ** *p* < 0.01, *** *p* < 0.001).

To examine the ability of potential antagonists to impair OA-activated signaling, measurements were carried out with the antagonists (10 μM) on a non-saturating concentration of OA in the CHO-K1 cells stably expressing each receptor. The reduction in cAMP level was quantified and normalized to the value achieved in the absence of antagonists (=100%). Chlorpromazine, cyproheptadine, metoclopramine, mianserin, phentolamine, methiothepin, epinastine, spiperone, SCH-23390, clozapine, asenapine, amitriptyline and doxepin displayed the antagonistic effects for each receptor, butaclamol, cinanerin and chlorprothixene inhibited the activation for CcOctβ2R and CcOctβ3R (Figure 5).

**Figure 6:**
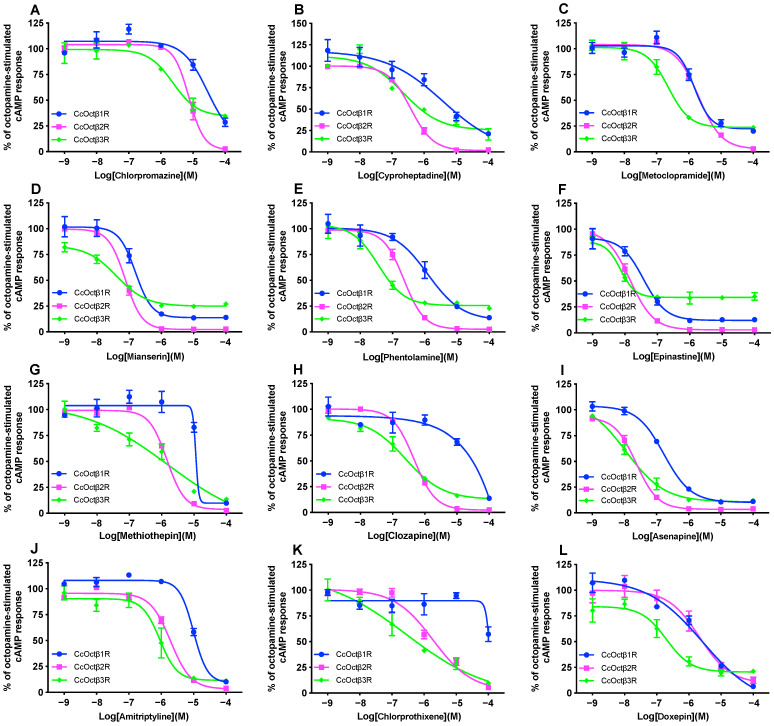
Dose-response curves of 12 antagonists on OA-stimulated intracellular cAMP production in CcOctβR-expressing CHO-K1 cells. Antagonists used were chlorpromazine (**A**), cyproheptadine (**B**), metoclopramide (**C**), mianserin (**D**), phentolamine (**E**), epinastine (**F**), methiothepin (**G**), clozapine (**H**), asenapine (**I**), amitriptyline (**J**), chlorprothixene (**K**), and doxepin (**L**). Each individual curve is normalized to its respective OA stimulation in the absence of antagonist (=100%). Data are presented as means ± SE of four experiments.

Next, the effects of 12 antagonists were measured with increasing concentrations on a non-saturating concentration of OA for each receptor (Figure 6). Ligand concentrations that resulted in the half-maximal inhibition of each receptor (IC_50_) were determined from the dose-response curves and shown in Table 2. However, IC_50_ values of methiothepin, clozapine and chlorprothixene for CcOctβ1R were not calculated (Table 2). Taking into account the effects of the antagonists on the basal activity, the rank order of antagonist potency was also different for each receptor. For CcOctβ1R, it was epinastine > mianserin > asenapine > phentolamine > metoclopramine > doxepin > cyproheptadine > amitriptyline > chlorpromazine. For CcOctβ2R, it was epinastine > asenapine > mianserin > phentolamine > cyproheptadine > clozapine > methiothepin > chlorprothixene > amitriptyline > metoclopramine > doxepin > chlorpromazine. For CcOctβ3R, it was epinastine > asenapine > phentolamine > mianserin > doxepin > cyproheptadine > metoclopramine > clozapine > chlorprothixene > amitriptyline > methiothepin > chlorpromazine. The most efficient antagonist on OA-induced CcOctβRs was epinastine, with IC_50_s ranging from ~7.34 × 10^−9^ M (CcOctβ3R) to ~3.76 × 10^−8^ M (CcOctβ1R) (Table 2). Asenapine and amitriptyline reduced OA-stimulated receptor activity by approximately 90% at the high concentrations for each receptor (Figure 6). For each antagonist, the order of receptor antagonistic efficacy was CcOctβ3R > CcOctβ2R > CcOctβ1R (Table 2).

## 3. Discussion

The availability of transcriptome and genome information provided an opportunity to study the physiological and behavioral roles of the insect octopaminergic signaling system. To meet this opportunity, determining the molecular and pharmacological data of OAR subtypes is an important step. OARs have been characterized in *D. melanogaster* [33,40,54], *Bombyx mori* [55,56], *Periplaneta americana* [57,58], *A. mellifera* [41,59,60], *N. lugens* [42,49], and *P. xylostella* [43,46,61,62]. Here, we cloned three OARs from *C. chilonis*, investigated their expression profiles, and pharmacologically characterized them.

CcOctβRs belong to the class A GPCR family, which are supported by D^3.49^RY motif at the cytoplasmic of TM3 and N^7.49^PLIY in TM7 (Supplementary Figures S1–S3). Most class A GPCRs are activated by ligands docking to specific amino acid residues in the receptor binding pocket close to the extracellular side [58], and functionally important residues of β-adrenergic-like OARs are highly conserved in CcOctβRs. The D^3.32^ residue in TM3 (D_121_ in CcOctβ1R, D_127_ in CcOctβ2R, D_75_ in CcOctβ3R), acts as an important residue that interacts with the protonated amino group of OA or TA. One of the closely grouped Ser residues (S^5.42^/^5.46^) in TM5 (S_210_SSVS_214_ in CcOctβ1R, S_216_SSIS_220_ in CcOctβ2R, S_168_SSVS_172_ in CcOctβ3R) can interact with the hydroxyl group of OA or TA [63,64]. The motif F^6.44^-X-X-C^6.47^-W^6.48^-X-P^6.50^-F^6.51^-F^6.52^ is highly conserved in TM6 of CcOctβRs. W^6.48^ and F^6.52^ residues were reported to interact with the ligand aromatic ring, which modulates the bend angle of TM6 near the well conserved kink at P^6.50^, prompting the cytoplasmic end of TM6 to move upon activation [65].

Our phylogenetic tree included major insect biogenic amine receptors and vertebrate adrenergic receptors. CcOctβRs were closely related to their orthologous receptors, and they clustered nicely with D1-like DARs and human β-adrenergic receptors (Figure 1), highlighting the concept of ‘ligand-hopping’ in the evolution of aminergic GPCRs [66]. In addition, pharmacological properties are similar in all these receptors, and activation of them induce an increase in intracellular cAMP production, suggesting that a close pharmacological and phylogenetic relationship occurs between OARs and DARs. Interestingly, they are four β-adrenergic-like OARs (Octβ1R-Octβ4R) reported in *A. mellifera* [41], and phylogenetic tree indicated that CcOctβ3R is more closely related to AmOctβ3R than AmOctβ4R (Figure 1). However, AmOctβ3R and AmOctβ4R were originated by alternative splicing of the same gene [41]. Additionally, the latest research showed that AmOctβ2R expressed multiple isoforms due to differential splicing [67]. Thus, these studies supported that β-adrenergic-like OAR family has only three genes and the different alternative splicing events may occur in the β-adrenergic-like OARs.

The transcriptional profiles of *CcOctβ1R*, *CcOctβ2R* and *CcOctβ3R* varied in different tissues (Figure 2). *CcOctβ1R* and *CcOctβ2R* were highly expressed in the head. Similar results were found in *PxOctβ1R* [62], *MsOctβ2R* [68], *DmOctβRs* [69], and *NlOctβRs* [42], which agreed with OA abundant in the central nervous system. *CcOctβ3R* was predominantly expressed in the antenna, and the *CcOctβ2R* transcript in the antenna was second only to that in the head. *BmOctβ2R* [35] and *NcOctβ2R* [70] were also expressed in the antenna. Interestingly, octopaminergic modulation in the antenna of honeybee workers could be enhanced not only by increasing the OA level, but also by upregulation of OARs [71], and OA has been reported to increase the olfactory responses of *Heliothis virescens* to female sex pheromones [72]. *CcOctβRs* were all expressed in the leg. The transcript levels of *NcOctβ2R* [70] and *TcOctβ2R* [50] were found to exhibit high expressions in the leg. There are large muscles in insect legs, and the expressions of *DmOctβRs* were all detected in the adult muscles [28]. OA regulates neuromuscular transmission, lipid oxidation and glycolysis for motor behavior [73].

CcOctβRs were stably expressed in CHO-K1 cells, which have been applied successfully to determine the pharmacological properties of biogenic amine receptors in previous studies; the cells that did not express the receptor showed no cAMP responses after the application of biogenic amines or synthetic antagonists [33,40,70]. Coupling of CcOctβRs to intracellular signaling cascades was measured, and it was found that they are coupled to G_s_ protein, and cause an elevation in cAMP production. All three receptors were activated by OA with EC_50_ values between 6.69 × 10^−9^ M (CcOctβ2R) and 3.90 × 10^−8^ M (CcOctβ1R) (Table 1). Thus, activation of CcOctβRs may be over a dynamic range of ligand concentrations. In *A. mellifera*, the orthologous receptors displayed EC_50_ values of 4.39 × 10^−8^ M (AmOctβ1R), 1.82 × 10^−9^ M (AmOctβ2R), and 3.30 × 10^−9^ M (AmOctβ3R) [41]. In *D. melanogaster*, EC_50_ values were 5.56 × 10^−9^ M (DmOctβ1R), 1.53 × 10^−8^ M (DmOctβ2R), and 1.40 × 10^−8^ M (DmOctβ3R) [40]. Besides OA, TA also activated all three CcOctβRs, and EC_50_s were shifted about two orders of magnitude to higher concentrations (Table 1). Similar results have been observed in DmOctβ1R [40], CsOctβ2R [52], BdOctβ1R [44], NcOctβ2R [70], and PaOctβ2R [58]. Interestingly, the previous studies indicated DA and dopaminergic agonists could activate OctβRs [44,50,70]. However, DA failed to activate CcOctβRs. In addition, CcOctβRs were also efficiently activated by naphazoline, DMPF, amitraz, lisuride, medetomidine, clonidine, and tolazoline. Similar observations have been shown in DmOctβRs [40], PxOctβRs [46,61,62], NlOctβ2R [49], and NcOctβ2R [70].

As a formamidine acaricide, amitraz is particularly effective against mites and ticks, as well as hemipteran and lepidopteran insects. Amitraz and its main metabolite DPMF act as high-affinity agonists of OARs [35]. For CcOctβRs, the rank order of the potency was DPMF > amitraz > OA (Table 1). Amitraz and DPMF could potently activate α- and β-adrenergic-like OARs of *B. mori*, and DPMF was more potent than amitraz and OA [35]. For PxOctβ1R and NcOctβ2R, amitraz and DPMF were less potent than OA [62,70]. The recent study indicated that amitraz and DPMF potently activated all four OARs from *Varroa destructor*, and VdOctβ2R was more sensitive to amitraz and DPMF than AmOctβ2R. Furthermore, behavioral assays using *D. melanogaster* OAR mutants identified Octβ2R as the sole target of amitraz [51].

Here, the interactions of three CcOctβRs with a series of potential antagonists were studied. Epinastine is an antagonist of vertebrate histamine H1 receptor, and also acts as an OAR antagonist in insects. Our results indicated epinastine was the most potent on CcOctβRs (Table 2), and similar observation was reported in NcOctβ2R and PaOctβ2R [58,70]. Mianserin is known as a non-selective 5-HT_2_ serotonin receptor antagonist in mammals but also a potent antagonist of OARs, TARs and DARs in insects [74,75]. Mianserin was highly potent to CcOctβRs (Table 2), and acted as the most efficient inhibitor for DmOctβRs and AmOctβRs [40,41]. In *A. mellifera*, the physiological and behavioral effects of mianserin have been studied, suggesting that it could affect gustatory and olfactory learning abilities [76,77]. Spiperone, SCH-23390, asenapine, amitriptyline and doxepin are generally known as markedly effective antagonists of DARs [78,79], and our results indicated that they also effectively blocked CcOctβRs (Figure 5 and Table 2), which may suggest that overlapping antagonistic interactions exist between DARs and OARs. Asenapine, amitriptyline and doxepin exhibited highly potent characteristics to DOP2, with nanomolar IC_50_ values for CqDOP2 and AaDOP2 [79]. The previous studies showed that phentolamine could efficiently block Octα1R, OctβR and Octα2R [40,57,60], and our results indicated that CcOctβRs were significantly attenuated by phentolamine (Figure 6), and also weakly activated by phentolamine (Supplementary Figure S4). Phentolamine is capable of showing agonist activity on OctβRs, and its dual actions were present in DmOctβRs [40], CsOctβ2R [52], and NcOctβ2R [70].

Overall, our study supports the proposition that OA signaling is highly complex in insects. Therefore, establishing pharmacological profiles of molecularly identified and heterologously expressed receptors is a valuable challenge, and by uncovering substances acting through individual receptors, it is possible to facilitate the design of novel in vivo pharmacological experiments which accurately address a specific physiological feature. This detailed pharmacological characterization of three CcOctβRs marks an important step towards precisely understanding the role of OA in this parasitoid.

## 4. Materials and Methods

### 4.1. Insects

Laboratory colony of *C. chilonis* has been continuously reared on its host *C. suppressalis*. They were initially obtained from rice fields of the experimental farm (Hangzhou, China). They were kept at a temperature of 28 ± 1 °C, a photoperiod of 16:8 h (L:D) and relative humidity of 75 ± 5%. After eclosion, the wasp adults were maintained in the glass containers and fed on 20% (*v/v*) honey solution [38,80].

### 4.2. Identification and Cloning of CcOctβR Genes

Transcriptome sequencing of *C. chilonis* was performed [80] and three putative β-adrenergic-like OARs (CcOctβ1R, CcOctβ2R and CcOctβ3R) were identified by BlastX (NCBI, Bethesda, MO, USA). Open reading frames were predicted via EditSeq (DNAstar, Madison, WI, USA).

The *C. chilonis* adults were sterilized with 75% ethanol, and their heads were then dissected into the TRIzol reagent (Invitrogen, Carlsbad, CA, USA). Total RNA was isolated according to the manufacturer’s protocol. The NanoDrop 2000 spectrophotometer (Thermo Fisher Scientific, Waltham, MA, USA) was used to determine the purities and concentrations of RNA samples. cDNA was synthesized with 1 μg RNA via TransScript One-Step gDNA Removal and cDNA Synthesis SuperMix kit (Transgen, Beijing, China). The full lengths of CcOctβRs were cloned by RT-PCR (Supplementary Table S2).

### 4.3. Sequence Alignment and Phylogenetic Analysis

The amino acid sequences of β-adrenergic-like OARs from *A. mellifera*, *D. melanogaster*, *C. suppressalis*, *N. lugens*, and *T. castaneum*, were obtained in the NCBI databases. Multiple sequence alignments were carried out using ClustalX2 and consequently edited with GeneDoc software. The putative transmembrane regions were predicted using TMHMM-2.0 (https://services.healthtech.dtu.dk/service.php?TMHMM-2.0, accessed on 15 August 2022). The putative PKC phosphorylation sites were identified by the NetPhos 3.1 Server (http://www.cbs.dtu.dk/services/NetPhos/, accessed on 15 August 2022). The potential N-glycosylation sites were predicted by the NetNGlyc 1.0 Server (http://www.cbs.dtu.dk/services/NetNGlyc/, accessed on 15 August 2022). To identify the potential orthologues of the cloned *C. chilonis* β-adrenergic-like OARs, the phylogenetic analysis of these receptors was performed with other insect biogenic amine receptors and human adrenergic receptors. The phylogenetic tree was constructed with MEGA 7.0 using the neighbor-joining method and 1000 bootstrap tests [81] and edited by the interactive tree of life (iTOL version 5) tool [82].

### 4.4. Expression Profiles of CcOctβRs

To investigate the relative expression levels of *CcOctβ1R*, *CcOctβ2R* and *CcOctβ3R* in various tissues of wasp adults, total RNAs from the heads without antennae, thorax, abdomen, antennae, and legs were extracted using the TRIzol reagent (Invitrogen, Carlsbad, CA, USA). The qRT-PCR primers were designed with Primer 3 (version 0.4.0, http://bioinfo.ut.ee/primer3-0.4.0/, accessed on 10 June 2022) (Supplementary Table S2). qRT-PCR analyses were conducted on a CFX 96 ^TM^ Real-Time Detection System (Bio-Rad, Hercules, CA, USA) with the following procedure: 95 °C for 30 s, and then 40 cycles of 95 °C for 5 s and 60 °C for 30 s with the melt curve dissociation step. qRT-PCR was done in a 25 μL volume containing 12.5 μL TB Green^®^ Premix Ex Taq^™^ II (Tli RNaseH Plus) (TaKaRa, Dalian, China), 1 μL each primer (10 μM), 2 μL cDNA, and 8.5 μL sterile H_2_O. Reactions for each sample were performed in triplicate. The *28S rRNA* was used as the reference gene to calculate the expression levels of *CcOctβ1R*, *CcOctβ2R* and *CcOctβ3R* [80,83], and the relative quantification was determined using the 2^−ΔΔCT^ method [84].

### 4.5. Construction of Expression Vectors

The eukaryotic expression vectors of CcOctβ1R, CcOctβ2R and CcOctβ3R were generated by RT-PCR using the specific primers (Supplementary Table S2), with the insertion of a Kozak consensus motif [85]. The KpnI and EcoRI were used to digest the pcDNA3.0 vector with hemagglutinin A tag (Invitrogen, Carlsbad, CA, USA), and the PCR products were then homologously cloned into pcDNA3.0 vector to obtain pcDNA3.0-CcOctβ1R, pcDNA3.0-CcOctβ2R and pcDNA3.0-CcOctβ3R with the ClonExpress^®^ II One Step Cloning Kit (Vazyme, Nanjing, China). All expression constructs were finally verified by DNA sequencing (Sangon Biotech, Shanghai, China). The high-quality expression plasmids were prepared with the EndoFree Plasmid Midi Kit (Qiagen, Hilden, Germany).

### 4.6. Heterologous Expression

Chinese hamster ovary K1 (CHO-K1) cells were cultured at 37 °C with 5% CO_2_ in an incubator. The culture medium was composed of DMEM/F-12 medium (Invitrogen, Carlsbad, CA, USA) and 10% fetal bovine serum (Invitrogen, Carlsbad, CA, USA). To prevent the contaminations of gram-positive and gram-negative bacteria, 100 units/mL penicillin and 100 mg/mL streptomycin (Invitrogen, Carlsbad, CA, USA) were added to the culture medium, respectively. The plasmids of pcDNA3.0-CcOctβ1R, pcDNA3.0-CcOctβ2R and pcDNA3.0-CcOctβ3R were transfected into CHO-K1 cells at the confluency of 70–90% in Petri dishes (3.5 cm). The Opti-MEM^®^ Reduced Serum Medium (1 mL) (Invitrogen, Carlsbad, CA, USA) containing 6 μL Lipofectamine^®^ 2000 (Invitrogen, Carlsbad, CA, USA) and 3 μg plasmid was prepared as transfection medium. After incubation for 20 min at room temperature, the transfection medium was supplemented after removing the growth medium. After 4–6 h in the incubator, the transfection medium was substituted with the fresh growth medium. Transfected cells were selected with the antibiotics G418 (800 μg/mL, Amresco, Solon, OH, USA) for 2–3 weeks, and G418-resistant cell clones were trypsin digested and transferred to cell culture plates (12-well) for expansion. The individual cell lines were monitored by immunocytochemistry for receptor expression, and then the stably transfected cells were cultured in the growth medium supplemented with 250 μg/mL G418.

### 4.7. cAMP Assays

Ligand stimulation of intracellular cAMP production was measured as described previously [33,70,78]. The cells were grown in 12-well plates (Nunc, Rochester, NY, USA) and cultured in the incubator. After 20 min of preincubation in the presence of 100 μM IBMX (Sigma-Aldrich, St. Louis, MO, USA) in D-PBS at 25 °C, the cells were added with 50 μL D-PBS (Invitrogen, Carlsbad, CA, USA) containing different concentrations of agonists, and they were then incubated for 20 min at 25 °C. The effects of antagonists were examined with 10 μM antagonists in the presence of the following OA concentrations: CcOctβ1R (100 nM), CcOctβ2R (30 nM) and CcOctβ3R (300 nM). After removing the solutions, the reactions were stopped by quickly adding 250 μL cell lysis buffer. The cell lysates were collected into the tubes and centrifuged at 12,000× *g* for 15 min. The cAMP Parameter Assay Kit (R&D Systems, Minneapolis, MN, USA) was used to determine cAMP in the supernatant. All tested compounds were dissolved in dimethyl sulfoxide, and further serially diluted with D-PBS.

### 4.8. Statistical Analysis

GraphPad Prism version 9.1.1 (GraphPad Software, San Diego, CA, USA) was used to analyze and display the data. Differences in transcript levels were analyzed by a one-way ANOVA followed by Tukey’s honestly significant difference (HSD) test. To examine whether statistical differences occurred in pharmacological comparisons, a one-way ANOVA followed by Dunnett’s multiple comparison test was performed (* *p* < 0.05, ** *p* < 0.01, *** *p* < 0.001). The EC_50_ and IC_50_ values were calculated using a special dose-response, nonlinear regression model.

## Figures and Tables

**Table 1:** EC_50_ and LogEC_50_ values for activation of cAMP response in CcOctβR-expressing CHO-K1 cells by various agonists.

Agonist	CcOctβ1R		CcOctβ2R		CcOctβ3R	
	EC_50_ (M)	LogEC_50_	EC_50_ (M)	LogEC_50_	EC_50_ (M)	LogEC_50_
OA	3.90 × 10^−8^	−7.41 ± 0.053	6.69 × 10^−9^	−8.18 ± 0.043	3.36 × 10^−8^	−7.47 ± 0.133
TA	1.53 × 10^−6^	−5.81 ± 0.080	7.59 × 10^−7^	−6.12 ± 0.062	2.99 × 10^−6^	−5.52 ± 0.039
Naphazoline	2.31 × 10^−10^	−9.64 ± 0.172	1.57 × 10^−11^	−10.8 ± 0.631	5.06 × 10^−9^	−8.30 ± 0.364
Clonidine	4.75 × 10^−7^	−6.32 ± 0.063	1.42 × 10^−7^	−6.85 ± 0.045	5.34 × 10^−7^	−6.27 ± 0.090
Tolazoline	1.30 × 10^−6^	−5.89 ± 0.078	4.85 × 10^−7^	−6.31 ± 0.027	5.04 × 10^−6^	−5.30 ± 0.082
Medetomidine	3.65 × 10^−7^	−6.44 ± 0.055	2.03 × 10^−8^	−7.69 ± 0.031	4.18 × 10^−8^	−7.38 ± 0.094
Lisuride	5.94 × 10^−8^	−7.23 ± 0.060	4.49 × 10^−8^	−7.35 ± 0.030	4.60 × 10^−9^	−8.34 ± 0.090
Amitraz	1.65 × 10^−8^	−7.78 ± 0.121	3.27 × 10^−9^	−8.49 ± 0.047	5.81 × 10^−9^	−8.24 ± 0.130
DPMF	9.25 × 10^−9^	−8.03 ± 0.078	2.37 × 10^−9^	−8.63 ± 0.046	4.35 × 10^−9^	−8.36 ± 0.089

**Table 2:** IC_50_ and LogIC_50_ values for inhibition of OA-induced cAMP response in CcOctβR-expressing CHO-K1 cells by various antagonists.

Antagonist	CcOctβ1R		CcOctβ2R		CcOctβ3R	
	IC_50_ (M)	LogIC_50_	IC_50_ (M)	LogIC_50_	IC_50_ (M)	LogIC_50_
Chlorpromazine	2.86 × 10^−5^	−4.54 ± 1.209	7.45 × 10^−6^	−5.13 ± 0.053	2.38 × 10^−6^	−5.62 ± 0.158
Cyproheptadine	3.95 × 10^−6^	−5.40 ± 0.483	3.80 × 10^−7^	−6.42 ± 0.024	2.31 × 10^−7^	−6.64 ± 0.261
Metoclopramide	1.48 × 10^−6^	−5.83 ± 0.134	2.03 × 10^−6^	−5.69 ± 0.048	2.32 × 10^−7^	−6.64 ± 0.084
Mianserin	1.57 × 10^−7^	−6.80 ± 0.080	7.58 × 10^−8^	−7.12 ± 0.022	4.06 × 10^−8^	−7.39 ± 0.094
Phentolamine	1.25 × 10^−6^	−5.91 ± 0.149	2.26 × 10^−7^	−6.65 ± 0.027	3.67 × 10^−8^	−7.44 ± 0.121
Epinastine	3.76 × 10^−8^	−7.43 ± 0.080	1.44 × 10^−8^	−7.84 ± 0.037	7.34 × 10^−9^	−8.13 ± 0.323
Methiothepin	n.a.	n.a.	1.47 × 10^−6^	−5.83 ± 0.043	1.63 × 10^−6^	−5.79 ± 0.734
Clozapine	n.a.	n.a.	4.69 × 10^−7^	−6.33 ± 0.024	2.55 × 10^−7^	−6.59 ± 0.089
Asenapine	1.70 × 10^−7^	−6.77 ± 0.036	2.34 × 10^−8^	−7.63 ± 0.029	1.13 × 10^−8^	−7.95 ± 0.119
Amitriptyline	9.92 × 10^−6^	−5.00 ± 0.029	1.95 × 10^−6^	−5.71 ± 0.064	9.03 × 10^−7^	−6.04 ± 0.090
Chlorprothixene	n.a.	n.a.	1.91 × 10^−6^	−5.72 ± 0.157	2.82 × 10^−7^	−6.55 ± 0.381
Doxepin	2.69 × 10^−6^	−5.57 ± 0.290	2.11 × 10^−6^	−5.68 ± 0.161	1.99 × 10^−7^	−6.70 ± 0.147

## Data Availability

Data are contained within the article or supplementary material.

## Supplementary Materials

The following supporting information can be downloaded at: https://www.mdpi.com/article/10.3390/ijms232314513/s1, Figure S1: Multiple sequence alignment of CcOctβ1R and its orthologous receptors from *Apis mellifera* (AmOctβ1R), *Chilo suppressalis* (CsOctβ1R), *Nilaparvata lugens* (NlOctβ1R), *Tribolium castaneum* (TcOctβ1R), and *Drosophila melanogaster* (DmOctβ1R); Figure S2: Multiple sequence alignment of CcOctβ2R and its orthologous receptors from *Apis mellifera* (AmOctβ2R), *Chilo suppressalis* (CsOctβ2R), *Nilaparvata lugens* (NlOctβ2R), *Tribolium castaneum* (TcOctβ2R), and *Drosophila melanogaster* (DmOctβ2R); Figure S3: Multiple sequence alignment of CcOctβ3R and its orthologous receptors from *Apis mellifera* (AmOctβ3R), *Nilaparvata lugens* (NlOctβ3R), *Tribolium castaneum* (TcOctβ3R), and *Drosophila melanogaster* (DmOctβ3R); Figure S4: Dose-response curves of phentolamine on intracellular cAMP levels in CHO-K1 cell lines stably expressing CcOctβRs; Table S1: The accession numbers of the sequences used in this study; Table S2: The primers used in this study.

## Abbreviations

OA	octopamine
TA	tyramine
DA	dopamine
5-HT	serotonin
HA	histamine
FK	forskolin
OAR	octopamine receptor
TAR	tyramine receptor
DAR	dopamine receptor
Octα1R	α_1_-adrenergic-like OARs
OctβR	β-adrenergic-like OARs
Octα2R	α_2_-adrenergic-like OARs
GPCR	G protein-coupled receptor
CHO-K1	Chinese hamster ovary K1
cAMP	cyclic adenosine monophosphate
IBMX	3-isobutyl-1-methylxanthine
D-PBS	Dulbecco’s phosphate-buffered saline
DMPF	N^2^-(2,4-dimethylphenyl)-N^1^-methyformamidine
TM	transmembrane
RT-PCR	reverse transcription-polymerase chain reaction
qRT-PCR	quantitative real-time polymerase chain reaction
ANOVA	analysis of variance
NCBI	National Center for Biotechnology Information
