# Supplementary material for: Molecular and Pharmacological Characterization of β-Adrenergic-like Octopamine Receptors in the Endoparasitoid Cotesia chilonis (Hymenoptera: Braconidae)
Source: Int J Mol Sci. 2022 Nov 22;23(23):14513. doi: 10.3390/ijms232314513 (PMC9740559; doi:10.3390/ijms232314513)
Supplement: Supplementary file 1 [file ijms-23-14513-s001.zip › ijms-1989056-supplementary.pdf]

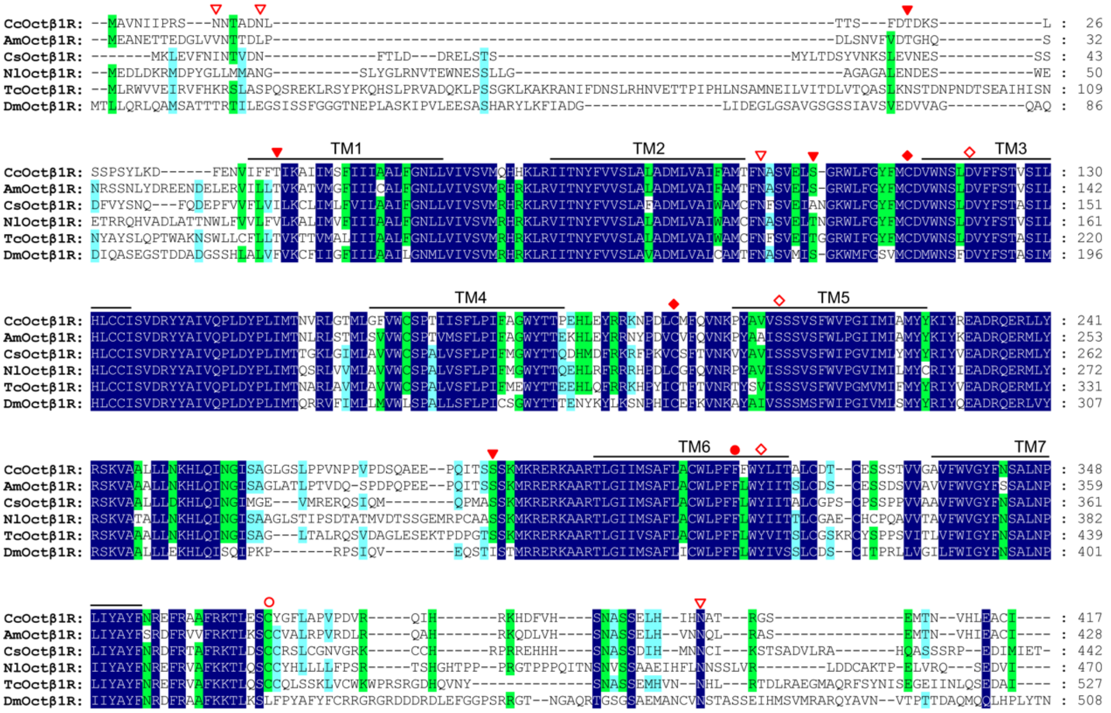

Figure S1. Multiple sequence alignment of CcOctβ1R and its orthologous receptors from *Apis mellifera* (AmOctβ1R), *Chilo suppressalis* (CsOctβ1R), *Nilaparvata lugens* (NlOctβ1R), *Tribolium castaneum* (TcOctβ1R), and *Drosophila melanogaster* (DmOctβ1R). Putative seven transmembrane (TM1-TM7) domains are indicated by black lines. Potential phosphorylation sites by PKC and potential N-glycosylation sites are marked with filled triangles and empty triangles, respectively. A disulphide bond is formed by two conserved cysteine residues that are labelled with diamonds. The empty diamonds mark the amino acid residues that are believed to function in ligand binding. The empty circles indicate potential palmitoylation sites. The filled circle labels the second phenylalanine (F_{314}) behind the FxxxWxP motif.

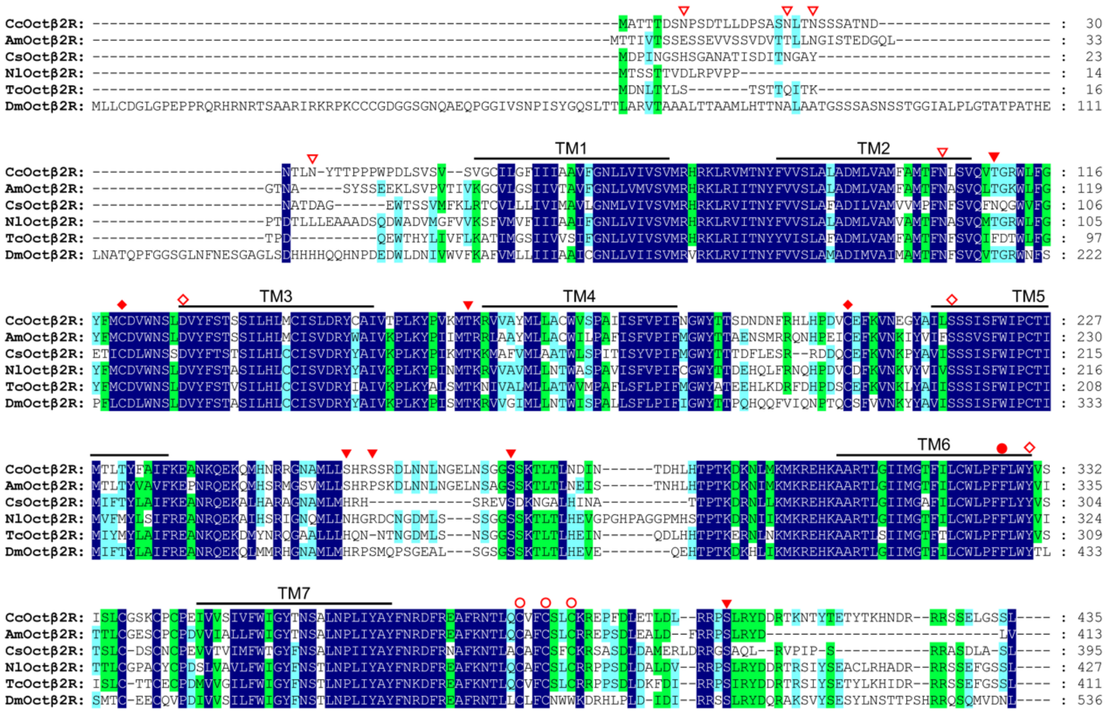

Figure S2. Multiple sequence alignment of CcOctβ2R and its orthologous receptors from *Apis mellifera* (AmOctβ2R), *Chilo suppressalis* (CsOctβ2R), *Nilaparvata lugens* (NlOctβ2R), *Tribolium castaneum* (TcOctβ2R), and *Drosophila melanogaster* (DmOctβ2R). Putative seven transmembrane (TM1-TM7) domains are indicated by black lines. Potential phosphorylation sites by PKC and potential N-glycosylation sites are marked with filled triangles and empty triangles, respectively. A disulphide bond is formed by two conserved cysteine residues that are labelled with diamonds. The empty diamonds mark the amino acid residues that are believed to function in ligand binding. The empty circles indicate potential palmitoylation sites. The filled circle labels the second phenylalanine (F_{327}) behind the FxxxWxP motif.

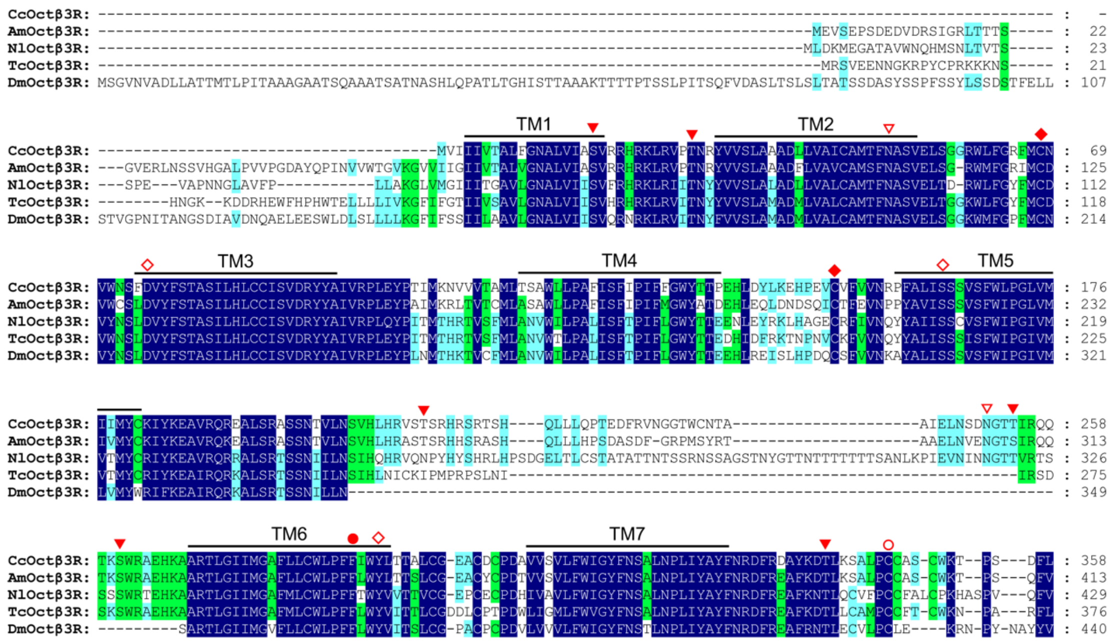

Figure S3. Multiple sequence alignment of CcOctβ3R and its orthologous receptors from *Apis mellifera* (AmOctβ3R), *Nilaparvata lugens* (NlOctβ3R), *Tribolium castaneum* (TcOctβ3R), and *Drosophila melanogaster* (DmOctβ3R). Putative seven transmembrane (TM1-TM7) domains are indicated by black lines. Potential phosphorylation sites by PKC and potential N-glycosylation sites are marked with filled triangles and empty triangles, respectively. A disulphide bond is formed by two conserved cysteine residues that are labelled with diamonds. The empty diamonds mark the amino acid residues that are believed to function in ligand binding. The empty circles indicate potential palmitoylation sites. The filled circle labels the second phenylalanine (F_{287}) behind the FxxxWxP motif.

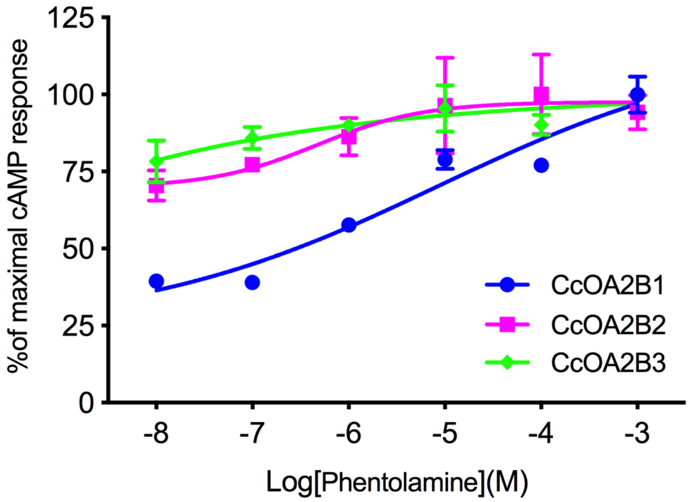

Figure S4. Dose-response curves of phentolamine on intracellular cAMP levels in CHO-K1 cell lines stably expressing CcOctβRs. The values were normalized to the maximal cAMP response (100%). The data represent means ± SE of four experiments.

Table S1. The accession numbers of the sequences used in this study.

Name	Accession No.	Description
**PROTOSTOMIA**, **Ecdysozoa**, Arthropoda		
CcOctβ1R	OP422531	octopamine receptor β1-R [*Cotesia chilonis*]
CcOctβ2R	OP422532	octopamine receptor β2-R [*Cotesia chilonis*]
CcOctβ3R	OP422533	octopamine receptor β3-R [*Cotesia chilonis*]
AmOctα1R	NP_001011565.1	octopamine receptor 1 [*Apis mellifera*]
AmOctβ1R	CCO13922.1	octopamine receptor β1-R [*Apis mellifera*]
AmOctβ2R	CCO13923.1	octopamine receptor β2-R [*Apis mellifera*]
AmOctβ3R	CCO13924.1	octopamine receptor β3-R [*Apis mellifera*]
AmOctβ4R	CCO13925.1	octopamine receptor β4-R [*Apis mellifera*]
AmOctα2R	XP_001122075.2	α2-adrenergic-like octopamine receptor [*Apis mellifera*]
AmDOP1	NP_001011595.1	dopamine receptor 1 [*Apis mellifera*]
AmDOP2	NP_001011567.1	dopamine receptor 2 [*Apis mellifera*]
AmDOP3	NP_001014983.1	dopamine receptor 3 [*Apis mellifera*]
AmDopEcR	AJE75857.1	dopamine/ecdysone receptor, partial [*Apis mellifera*]
AmTA1	NP_001011594.1	tyramine receptor type 1 [*Apis mellifera*]
AmTA2	NP_001032395.1	tyramine receptor type 2 [*Apis mellifera*]
CsOctα1R	AEQ33589.1	octopamine receptor 1[*Chilo suppressalis*]
CsOctβ1R	AGV79326.1	octopamine receptor β1-R [*Chilo suppressalis*]
CsOctβ2R	AEO89318.1	octopamine receptor β2-R [*Chilo suppressalis*]
CsOctα2R	AIC75370.1	α2-adrenergic-like octopamine receptor long variant [*Chilo suppressalis*]
CsDOP1	AKR18178.1	dopamine receptor 1 [*Chilo suppressalis*]
CsDOP2	AKR18179.1	dopamine receptor 2 [*Chilo suppressalis*]
CsDOP3	AKR18180.1	dopamine receptor 3 [*Chilo suppressalis*]
CsTA1	AFG26689.1	tyramine receptor type 1 [*Chilo suppressalis*]
CsTA2	ADK91078.1	tyramine receptor type 2 [*Chilo suppressalis*]
DmOctα1AR	NP_732541.1	octopamine receptor in mushroom bodies, isoform F [*Drosophila melanogaster*]
DmOctα1BR	NP_524669.2	octopamine receptor in mushroom bodies, isoform B [*Drosophila melanogaster*]
DmOctβ1R	NP_651057.1	octopamine receptor β1-R [*Drosophila melanogaster*]
DmOctβ2R	NP_001034049.1	octopamine receptor β2-R [*Drosophila melanogaster*]
DmOctβ3R	NP_001034043.2	octopamine receptor β3-R [*Drosophila melanogaster*]
DmOctα2R	NP_650754.2	α2-adrenergic-like octopamine receptor [*Drosophila melanogaster*]
DmDOP1	CAA54451.1	dopamine receptor 1 [*Drosophila melanogaster*]
DmDOP2	NP_733299.1	dopamine receptor 2 [*Drosophila melanogaster*]
DmDOP3	AAN15955.1	dopamine receptor 3 [*Drosophila melanogaster*]
DmDopEcR	AAF47893.1	dopamine/ecdysteroid receptor [*Drosophila melanogaster*]
DmTA1	NP_524419.2	tyramine receptor type 1 [*Drosophila melanogaster*]
DmTA2	NP_650652.1	tyramine receptor type 2 [*Drosophila melanogaster*]
NlOctα1R	ATY68965.1	octopamine receptor 1 [*Nilaparvata lugens*]
NlOctβ1R	ATY68966.1	octopamine receptor β1-R [*Nilaparvata lugens*]

NlOctβ2R	ASA47149.1	octopamine receptor β2-R [*Nilaparvata lugens*]
NlOctβ3R	QBC74627.1	octopamine receptor β3-R [*Nilaparvata lugens*]
NlOctα2R	ATY68968.1	α2-adrenergic-like octopamine receptor long variant [*Nilaparvata lugens*]
TcOctβ1R	NP_001280514.1	octopamine receptor β1-R [*Tribolium castaneum*]
TcOctβ2R	NP_001280501.1	octopamine receptor β2-R [*Tribolium castaneum*]
TcOctβ3R	NP_001280505.1	octopamine receptor β3-R [*Tribolium castaneum*]
TcDopEcR	NP_001280528.1	dopamine/ecdysteroid receptor [*Tribolium castaneum*]
DmFR	NP_647758.1	FMRFamide receptor [*Drosophila melanogaster*]
DmninaE	AAF55712.1	neither inactivation nor afterpotential E [*Drosophila melanogaster*]
**PROTOSTOMIA**, **Ecdysozoa**, Priapulida		
Pcα1	XP_014662992.1	α_{1A}-adrenergic receptor-like [*Priapulus caudatus*]
Pcα2	XP_014681069.1	α_{2C}-adrenergic receptor-like [*Priapulus caudatus*]
**PROTOSTOMIA**, **Lophotrochozoa**, Annelida		
Pdα1	APC23842.1	α_1-adrenergic receptor [*Platynereis dumerilii*]
Pdα2	APC23843.1	α_2-adrenergic receptor [*Platynereis dumerilii*]
PdTA1	AKQ63052.1	tyramine receptor 1 [*Platynereis dumerilii*]
PdTA2	APC23184.1	tyramine receptor 2 [*Platynereis dumerilii*]
PdOctα1R	APC23183.1	octopamine receptor 1 [*Platynereis dumerilii*]
PdOctβR	APC23841.1	octopamine receptor 2 [*Platynereis dumerilii*]
**DEUTEROSTOMIA**, **Ambulacraria**, Priapulida		
Skα1	ALR88680.1	α_1-adrenergic receptor-like 067 [*Saccoglossus kowalevskii*]
Skα2	XP_002734932.1	α_{2C}-adrenergic receptor-like [*Saccoglossus kowalevskii*]
SkTA1	XP_002742354.2	tyramine receptor 1 [*Saccoglossus kowalevskii*]
SkTA2A	XP_002734062.1	tyramine receptor 2A [*Saccoglossus kowalevskii*]
SkTA2B	XP_006812999.1	tyramine receptor 2B [*Saccoglossus kowalevskii*]
SkOctα1R	XP_006823182.1	α_1-adrenergic-like octopamine receptor [*Saccoglossus kowalevskii*]
SkOctβR	XP_002733926.1	β-adrenergic-like octopamine receptor [*Saccoglossus kowalevskii*]
**DEUTEROSTOMIA**, **Chordata**, Vertebrata		
Hsβ1	NP_000675.1	β-1 adrenergic receptor [*Homo sapiens*]
Hsβ2	NP_000015.1	β-2 adrenergic receptor [*Homo sapiens*]
Hsβ3	NP_000016.1	β-3 adrenergic receptor [*Homo sapiens*]
Hsα1A	NP_000671.2	α-1A adrenergic receptor [*Homo sapiens*]
Hsα1B	NP_000670.1	α-1B adrenergic receptor [*Homo sapiens*]
Hsα1D	NP_000669.1	α-1D adrenergic receptor [*Homo sapiens*]
Hsα2A	NP_000672.3	α-2A adrenergic receptor [*Homo sapiens*]
Hsα2B	NP_000673.2	α-2B adrenergic receptor [*Homo sapiens*]
Hsα2C	NP_000674.2	α-2C adrenergic receptor [*Homo sapiens*]
HsD1	NP_000785.1	D(1A) dopamine receptor [*Homo sapiens*]
HsD2	NP_000786.1	D(2) dopamine receptor isoform long [*Homo sapiens*]
HsD3	NP_000787.2	D(3) dopamine receptor isoform a [*Homo sapiens*]
HsD4	NP_000788.2	D(4) dopamine receptor [*Homo sapiens*]
HsD5	NP_000789.1	D(1B) dopamine receptor [*Homo sapiens*]

Table S2. The primers used in this study.

Primer name	Sequence (5'-3')
**For cDNA cloning**	
CcOctβ1R-compF	CTTAAAGGTTCTCCATCACAAGACG
CcOctβ1R-compR	CTATATTTAAATACACGCCTCCAAG
CcOctβ2R-compF	AGCTGCGTCACAAGTCGTCGTCGTC
CcOctβ2R-compR	GTTTCGTTTCAGAGACTGCTTCC
CcOctβ3R-compF	CGATGAAAGACAGGAATCAGCGTC
CcOctβ3R-compR	GAATACAAAGTCATTCCGCGCGAGC
**For qRT-PCR**	
CcOctβ1R-RTF	TTTTGGGTGCCAGGAATCAT
CcOctβ1R-RTR	TCCTAAGCCCGCTGATATGC
CcOctβ2R-RTF	TCGTCAGCATTGTCTTCTGG
CcOctβ2R-RTR	TCGATCGTTGTGTTTGGTGT
CcOctβ3R-RTF	AGCAGCCGCTGATTTATTGG
CcOctβ3R-RTR	GCTGATGCAGCAAAGGTGAA
28S rRNA-RTF	ACGTGCAAATCGATCGTCTG
28S rRNA-RTR	CAAGCCAGAGATCTCACCCA
**For eukaryotic expression**	
pcDNA3.0-CcOctβ1R-KpnI-F	TATAGGGAGACCCAAGCTTGGTACC***GCCACC***ATGG<br>CAGTGAATATAATTCCC
pcDNA3.0-CcOctβ1R-EcoRI-R	AGTGTGATGGATATCTGCA***GAATTC***TTAAATAC<br>ACGCCTCCAAGTG
pcDNA3.0-CcOctβ2R-KpnI-F	TATAGGGAGACCCAAGCTT<br>GGTACC***GCCACC***ATGGCGACGACAACGGATTC
pcDNA3.0-CcOctβ2R-EcoRI-R	AGTGTGATGGATATCTGCA***GAATTC***TCAGAGACTGC<br>TTCCTAATTC
pcDNA3.0-CcOctβ3R-KpnI-F	TATAGGGAGACCCAAGCTT<br>GGTACC***GCCACC***ATGGTTATTATTATAGTTACC
pcDNA3.0-CcOctβ3R-EcoRI-R	AGTGTGATGGATATCTGCA***GAATTC***CTATAGAA<br>AATCTGAGGGCG
